# Comment/response to letter to editor

**DOI:** 10.1080/2090598X.2021.1902128

**Published:** 2021-03-18

**Authors:** Elias Ayoub

**Affiliations:** Urology, La Pitie Salpetriere Hospital, Paris, France

Professor,

I want to thank you for your interesting and precious comments. I understand that the technique described in this study includes some steps that could cause harm to patients if not done by an experienced surgeon in a tertiary referral centre where all complications could be dealt with.

We intended to highlight these conclusions several time in the research. More training and prospective studies should be done before considering any generalisation of this or any other fluoroless technique.

Respectfully,

